# Idiopathic Atrophoderma of Pasini and Pierini: Response to Mycophenolic Acid Treatment

**DOI:** 10.7759/cureus.77439

**Published:** 2025-01-14

**Authors:** Monica Ceballos-Pérez, Ilse Marilu Gutierrez Villarreal, Ivan E Cano Lizarraga, Diego Emilio Gómez López, Marlene Y Solís Ramírez, Grecia Cantu, Circe Ancona Castro

**Affiliations:** 1 Department of Dermatology, Instituto de Seguridad y Servicios Sociales de los Trabajadores del Estado (ISSSTE) Monterrey Regional Hospital, Monterrey, MEX; 2 Department of General Practice, Tecnológico de Monterrey, Monterrey, MEX

**Keywords:** atrophoderma of pasini and pierini, dermal atrophy, mycophenolic acid, non-indurated dermatosis, therapeutic approach

## Abstract

Atrophoderma of Pasini and Pierini (APP) is a rare skin condition of unknown etiology. It is characterized by dermal atrophy with single or multiple hyperpigmented, non-indurated, and slightly depressed plaques on the trunk or limbs; unlike morphea, this condition does not cause induration.

In this report, we analyze the case of a patient diagnosed with APP with a 19-year history of progression, aiming to highlight the few cases published in the literature. The patient responded favorably to treatment with mycophenolic acid (MPA).

Therapeutic approaches for this condition are controversial due to its long-term progression and low frequency. Currently, there are no published reports on the use of MPA for the treatment of this condition.

## Introduction

Atrophoderma of Pasini and Pierini (APP) is a rare cutaneous condition, initially described by Pasini in 1923 under the name "progressive idiopathic atrophoderma"; in 1936, Vivoli described it again, suggesting an association with morphea, later on, it was associated by other authors with Borrelia Burgdorferi, although its etiology is still not well established [1].

APP predominantly affects women, causing dermal atrophy presenting as single or multiple well-defined plaques described as "cliff-edge borders" or "footprints in the snow," with no induration. It is usually asymptomatic and progressive, initially appearing on the trunk and subsequently spreading to the chest, arms, and abdomen. Histopathologically, cutaneous appendages are preserved, aiding in its differentiation from morphea, its main differential diagnosis [2].

Regarding the prognosis of APP, it tends to progress over 10 to 20 years without systemic involvement, and although the disease may not progress further, existing lesions do not regress, making treatment aimed at halting progression essential [3]. 

## Case presentation

This is the case of a 46-year-old female patient with no significant personal medical history relevant to the current condition. Her symptoms began in February 2004, initially with the appearance of a "patch" in the upper left quadrant of the abdomen, accompanied by mild tenderness to palpation. She was initially treated by a private practitioner with low-potency topical steroids and keratolytics with salicylic acid 3% for six months. Subsequently, she remained untreated for 18 years, with a slow and progressive evolution of the dermatosis.

**Figure 1:**
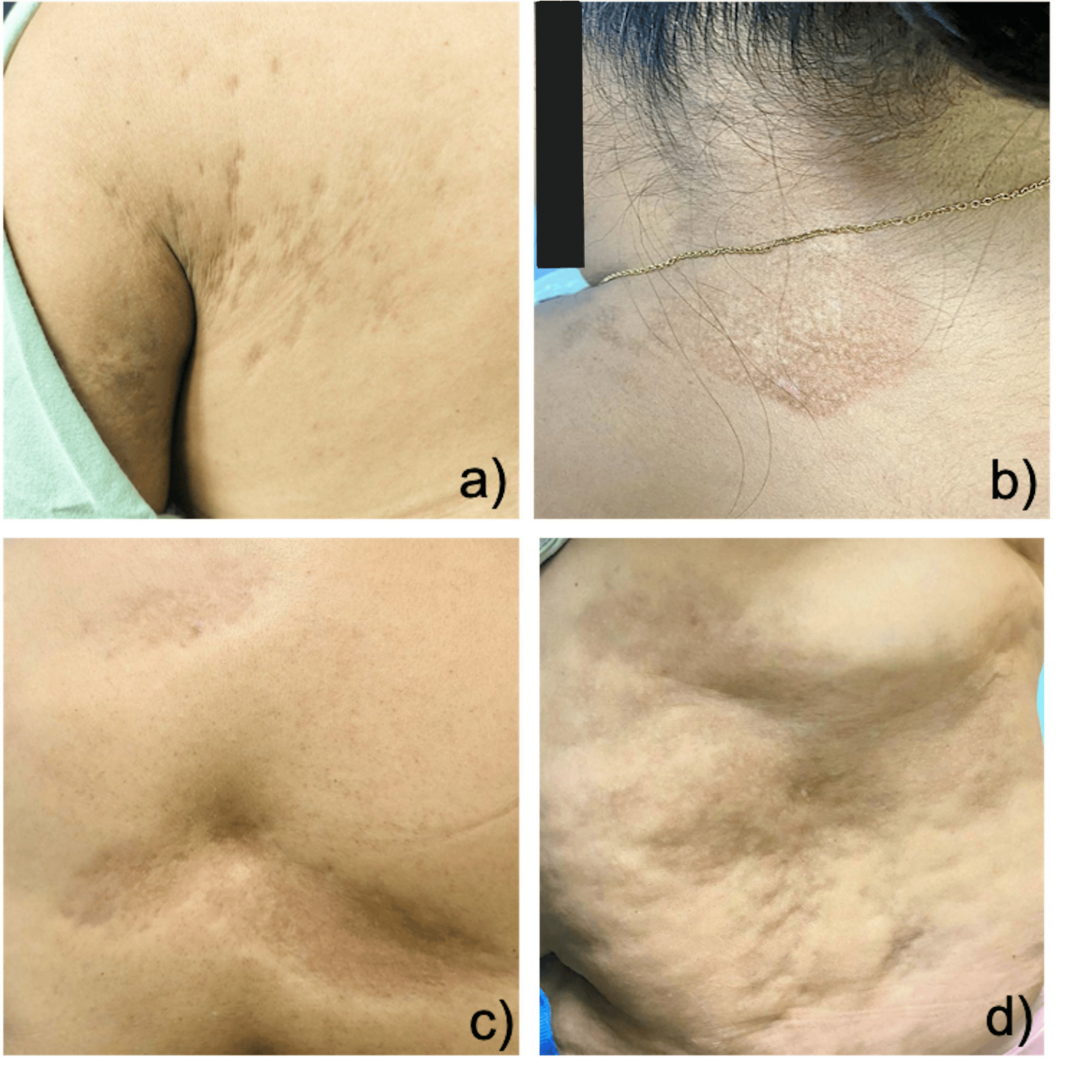
Dermatoses that affect a, b, and c) posterior trunk, which affects the left thoracic and lumbar region, d) as well as the abdomen in left quadrates, characterized by multiple plaques of 5 to 10 mm that in some areas tend to converge forming plaques of up to 20cm in diameter, atrophic, oval in shape, erythematous-brown in color, with depressed “cliff” edges.

In March 2022, she came to our clinic with a disseminated dermatosis involving the left upper extremity: affecting the inner aspect of the arm, posterior trunk which involved the left thoracic and lumbar regions, left quadrant of the abdomen, left lower extremity involving the thigh and posterior-lateral aspect of the leg. The lesions were characterized by multiple plaques ranging in size from 5 to 10 mm with depressed "cliff-edge" borders (Figure 1).

**Figure 2:**
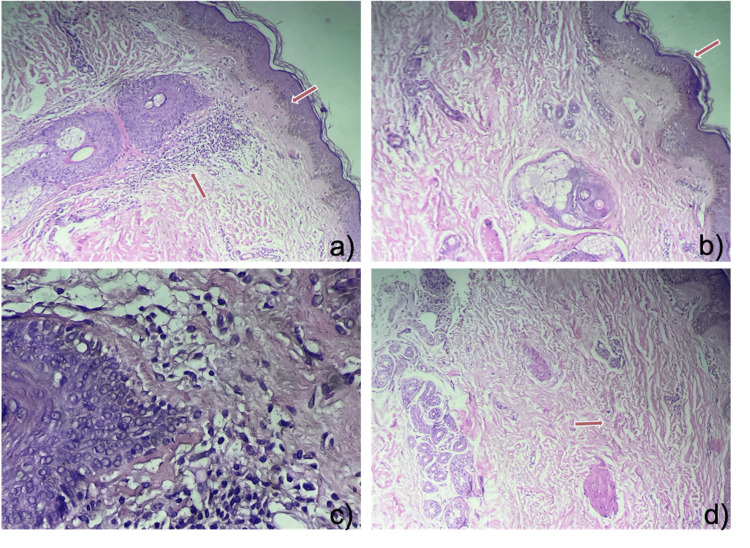
Skin biopsy using a 3mm punch with Hematoxylin and Eosin staining. In the upper sections, a panoramic view shows a) Epidermis with hyperpigmentation of the basal layer and b) mild orthokeratotic hyperkeratosis. In the lower sections at 40x magnification, c) and d) in the dermis, there is mild perivascular, perifollicular, and periadnexal infiltrate predominantly composed of lymphocytes, with preserved appendages.

Due to the suspicion of APP, laboratories were requested, in search of an associated etiology, which were reported within normal parameters with the exception of altered fasting glucose and dyslipidemia. Biopsy was also performed showing changes suggestive of APP (Figure 2).

**Figure 3:**
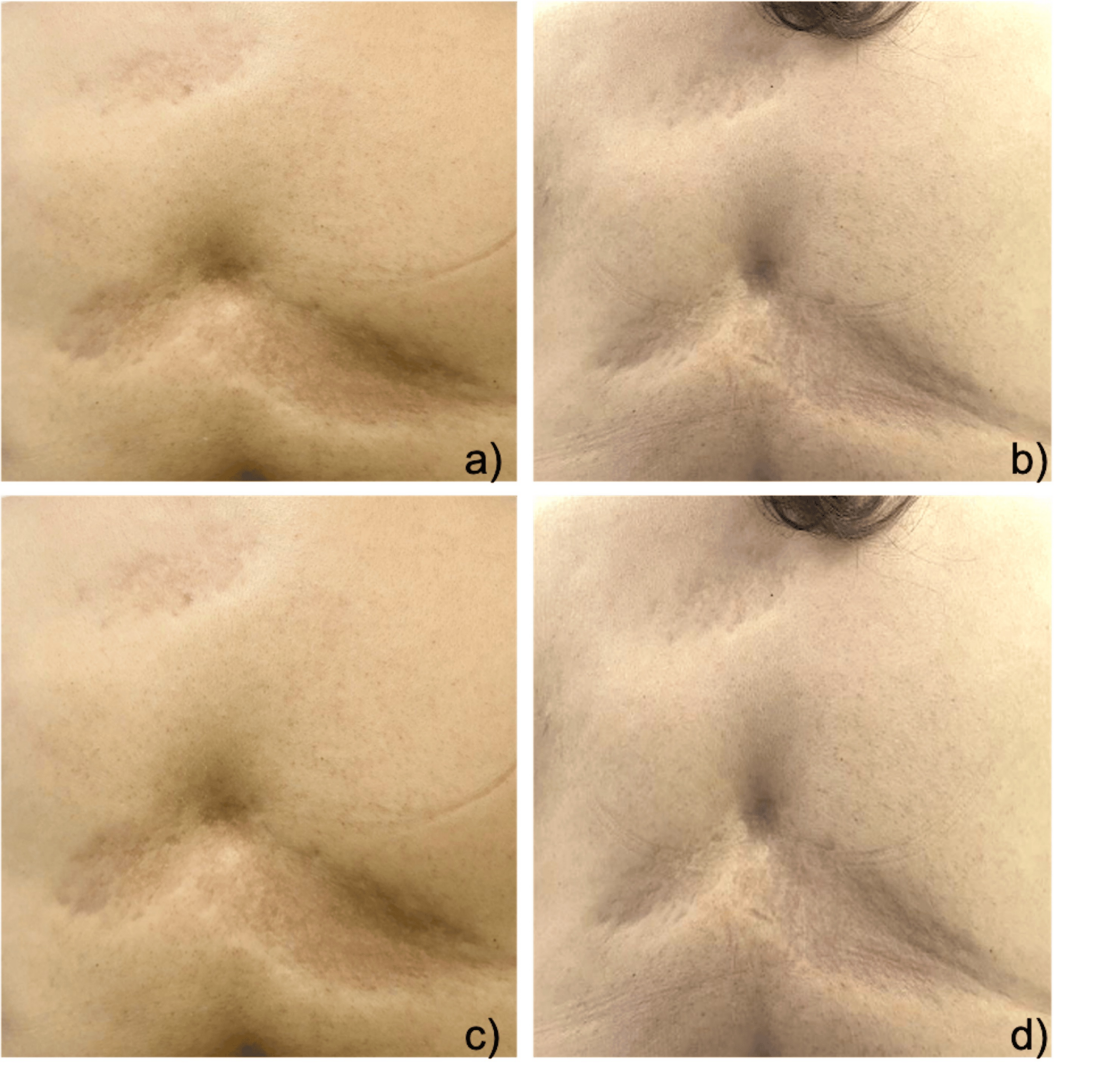
Posterior thoracic region at the dorsal level: a) prior to medical treatment and b) after medical treatment showing remission of erythema, attenuation of "depression" and "cliff edges", similarly observed in the abdominal region in the left quadrant: c) prior to medical treatment and d) after medical treatment.

Given the progressive nature, systemic treatment was initiated with mycophenolic acid 500 mg every 12 hours, in addition to topical pimecrolimus every 12 hours. Two months after starting treatment, new lesions appeared on the left forearm and leg; therefore, we decided to increase the mycophenolic acid (MPA) dose to 1000 mg every eight hours, along with a cycle of high-potency topical steroids. There were no new lesions or increase in the size of existing lesions in the following six months after treatment adjustment (Figure 3).

## Discussion

Currently, there are fewer than 200 cases of APP published in the literature, with one reported in México in 1999 [1,2]. 

APP has been associated with several pathologies and based on these are the treatments that are currently reported in the literature. This entity has been associated with infection by Borrelia Burgdorferi, which responds adequately to treatment with penicillin or tetracycline with remission of the dermatosis [2]. Likewise, there are cases associated with lupus or psoriasis with a good response to management with topical steroids, topical calcineurin inhibitors, and antimalarials such as hydroxychloroquine and methotrexate. There are also isolated reports of the use of a Q-switch laser with good response in hyperpigmented lesions [4,5]. 

Some authors associate APP with morphea, considering it a distinct variant both clinically and histopathologically. In this case, treatment with MPA was chosen. MPA is an immunosuppressive agent whose mechanism of action is related to its effects on the immune system and cellular proliferation. By modulating the immune response, it benefits inflammatory skin conditions and helps reduce excessive immune activation that may contribute to skin changes. While the exact mechanism in APP is not clearly established, MPA’s general immunosuppressive and anti-inflammatory properties might help prevent further progression of dermal atrophy or improve the condition. MPA has been widely used and described in morphea and systemic sclerosis, where it is considered a first-line treatment, particularly in patients with lung disease. Recent studies, however, have also shown a good response in skin involvement, with softening of the lesions [6-10]. 

In our patient, in addition to stopping the progression of the dermatosis, it helped mitigate the presence of erythema and edema, data suggestive of APP activity.

Due to the low incidence of this pathology, there is no standardized treatment, and to our knowledge, there is no reported case of APP that has been managed with MPA.

## Conclusions

This case describes a patient with long-standing APP, who showed a positive response to treatment with MPA, which is an unexplored therapeutic approach in this disease. This is combined with the few reported cases of APP. This report and review of the literature contribute to the understanding of this dermatosis, as well as to substantiate the demonstrated effectiveness of this treatment in other skin diseases with which APP is associated, such as morphea, and its ability to stop the progression of lesions and symptoms associated with APP activity.

In summary, the successful treatment with MPA highlights the imperative need to explore innovative therapies in the management of APP, especially considering the lack of standardized options due to its low incidence and the absence of previous reports on the use of this medication in this specific disease.
